# Visual Impairments Are Associated With Retinal Microvascular Density in Patients With Parkinson’s Disease

**DOI:** 10.3389/fnins.2021.718820

**Published:** 2021-08-12

**Authors:** Min Zhou, Lei Wu, Qinyuan Hu, Congyao Wang, Jiacheng Ye, Tingting Chen, Pengxia Wan

**Affiliations:** ^1^Department of Ophthalmology, The First Affiliated Hospital, Sun Yat-sen University, Guangzhou, China; ^2^Department of Neurology, The First Affiliated Hospital, Sun Yat-sen University, Guangzhou, China

**Keywords:** visual impairment, retinal microvascular density, Parkinson’s disease, optical coherence tomography angiography, visual evoked potential

## Abstract

**Objective:**

This study aimed to evaluate retinal microvascular density in patients with Parkinson’s disease (PD) and its correlation with visual impairment.

**Methods:**

This cross-sectional study included 24 eyes of 24 patients with PD and 23 eyes of 23 healthy controls. All participants underwent ophthalmic examination, visual evoked potential (VEP) test, 25-item National Eye Institute Visual Function Questionnaire (NEI VFQ-25), and optical coherence tomography angiography (OCTA) examination. The correlation between retinal microvascular density and visual parameter was evaluated using Spearman correlation analysis, and the area under receiver operating characteristic curve (AUROC) was calculated.

**Results:**

Parkinson’s disease patients had prolonged P100 latency (*P* = 0.041), worse vision-related quality of life (composite score and 3 of 12 subscales in NEI VFQ-25), and decreased vessel density (VD) in all sectors of 3-mm-diameter region (all *P* < 0.05) compared with healthy controls. There were no statistical differences in the ganglion cell-inner plexiform layer (GCIPL) thickness and retinal nerve fiber layer (RNFL) thickness between the two groups. A negative correlation was found between P100 latency and nasal and superior sectors of macular VD in a 3-mm-diameter region (*r* = −0.328, *P* = 0.030; *r* = −0.302, and *P* = 0.047, respectively). Macular VD in a 3-mm-diameter region showed diagnostic capacities to distinguish PD patients from healthy controls (AUROCs, ranging from 0.655 to 0.723).

**Conclusion:**

This study demonstrated that decreased retinal microvascular density was correlated with visual impairment in PD patients. Retinal microvasculature change may occur earlier than visual decline and retinal structure change and has the potential to be a promising diagnostic marker for early PD.

## Introduction

Parkinson’s disease (PD) is the second most common neurodegenerative disease characterized by a wide range of motor and non-motor symptoms (Lee and Gilbert, 2016). Visual symptoms together with other non-motor disorders such as cognitive deficits, hyposmia, and gastrointestinal dysfunctions were widely recognized to affect the life quality of PD patients and may occur several years before the onset of cardinal motor signs (Berg et al., 2015; Mahlknecht et al., 2015). Studies have reported as high as 78% of PD patients had at least one visual symptom, such as reading difficulties, double vision, and misjudgment of objects and distances (Archibald et al., 2011; Urwyler et al., 2014). The impact of visual disorders is particularly annoying for patients with PD, because they have impairments in control of movement and postural stability, and which could be compensated through visual guidance (Azulay et al., 1999; Davidsdottir et al., 2005).

The underlying pathogenesis of these visual disorders is regarded as relevant with α-synuclein deposition (Bodis-Wollner et al., 2014) and dopamine deficiency (Harnois and Di Paolo, 1990) in the retina, and similar to the pathological features of PD in the brain. Recently, vessel degeneration has been considered to be an additional factor contributing to the progression of PD. In postmortem brain tissue of PD patients, capillaries were shorter in average length, were less in number, and had fewer branches those in age-matched controls (Guan et al., 2013). And decreased cerebral blood flow has also been found in non-demented patients with PD, suggesting that perfusion abnormality may be a potential predictor upstream of cognitive impairment and neurodegeneration (Syrimi et al., 2017). However, the detections for brain vasculature are expensive and time-consuming. As a constituent of the central nervous system (CNS), the retina shows a striking resemblance to the brain and spinal cord. The cellular and molecular mechanisms implicated in retinal neurodegenerative processes are similar to those in the CNS (London et al., 2013; Maresca et al., 2013).

Therefore, the retina can serve as a window to observe microcirculation in the brain. Optical coherence tomography angiography (OCTA) is a functional extension of optical coherence tomography (OCT) imaging that facilitates the visualization of microvascular and morphological structure non-invasively in the retina (Spaide et al., 2018). Recently, several research findings described the decreased retinal microvascular density in patients with PD, which can serve as a surrogate biomarker for the diagnosis of PD (Kwapong et al., 2018; Zou et al., 2020). However, studies focusing on whether altered retinal microvasculature affects visual function were scarce.

Thus, the aim of our study was to determine the microvasculature alterations in the retina and its relationship with visual function in patients with PD.

## Materials and Methods

### Participants

The protocol of this study was approved by the Ethics Committee of the First Affiliated Hospital of Sun Yat-sen University. Participants provided informed written consent, and the tenets of the Declaration of Helsinki were followed throughout. Consecutive patients were recruited from the neurology outpatient clinic of the First Affiliated Hospital of Sun Yat-sen University, and healthy subjects were recruited from the patients’ non-consanguineous families or friends *via* asking for their willingness to participate. Idiopathic PD was defined by an experienced neurologist based on the United Kingdom Brain Bank criteria (Reichmann, 2010), and medical records including the duration of disease and treatment were carefully collected. Eligible patients were aged 40 years or older and only received drug treatment without any surgical intervention (e.g., deep brain stimulation treatment). The exclusion criteria were as follows: patients with psychiatric or neurological diseases other than PD, such as dementia or multiple sclerosis; diabetes, uncontrolled hypertension, or other systemic diseases which could affect the visual system; history of ocular trauma or surgery; family history of glaucoma; high refractive error (± 6.00D spherical equivalent); intraocular pressure (IOP) > 21 mmHg; media opacifications; concomitant ocular diseases such as corneal disease, glaucoma, or retinal disease. After preliminary screening, patients were asked to refrain from drug administration the night before, and on the day of examination. Each participant was first scored clinically and neuropsychologically by the neurologist and subsequently examined by the ophthalmologists using visual evoked potential (VEP), and OCTA. Clinicians were blind to each other’s results during the assessment.

### Neuropsychological and Clinical Assessments

All patients were evaluated for cognitive function and disease severity by the same experienced neurologist (LW). Cognitive function was assessed using the Mini-Mental State Examination (MMSE). MMSE is a 30-point questionnaire that assesses orientation, memory, attention, language, and visuospatial ability, and scores < 27 points are indicative of likely cognitive impairment (Folstein et al., 1975). Disease severity was evaluated using the Unified Parkinson’s Disease Rating Scale III (UPDRS III) (Goetz et al., 2008) and Hoehn and Yahr (1967) [H&Y] stage. Patients were assessed in the “off” state before the regular dose of the drug.

### Ophthalmologic Examination

All participants received a complete ophthalmic examination, including best-corrected visual acuity (BCVA), IOP, and examination of the anterior segment, and fundus by an experienced ophthalmologist. Vision-related quality of life was assessed using the 25-item National Eye Institute Visual Function Questionnaire (NEI VFQ-25). After the NEI VFQ-25 was administered, scores were recorded according to the guideline provided. Scores range from 0 to 100, with higher scores indicating better visual function (Mangione et al., 2001). All subjects were evaluated with VEP (MKWHAMD, CN-V1.4, Huzhou Medconova Medical Technology Co., Ltd., Huzhou, China) in a dark and quiet room. Stimulation was monocular after covering the other eye, and visual stimuli followed a checkerboard pattern.

### Optical Coherence Tomography Angiography

The imaging of all subjects was performed using the Zeiss Cirrus HD-OCT 5000 with an AngioPlex OCTA instrument (Cirrus; Zeiss, Dublin, CA, United States). A standard 3 × 3 mm scan was performed centered on the fovea, while the 6 × 6 mm scan was performed centered on the optic nerve head (ONH) as well as fovea. Vessel density (VD) (defined as the total length of the perfused vasculature per unit area in the region of measurement) of the superficial capillary plexus (SCP) (from the layer of the inner limiting membrane to the inner plexiform layer) was measured automatically in all 3 × 3 mm and 6 × 6 mm scans. Images of VD were calculated separately at various distances from the fovea: central (1-mm-diameter region), inner ring (1–3-mm-diameter region), outer ring (3–6-mm-diameter region), and full area (6-mm-diameter region). Furthermore, inner-ring and outer-ring regions were divided into four quadrants. The foveal avascular zone (FAZ) was assessed automatically in the 3 × 3 mm scan. The central subfield thickness (CST) (from the layer of the inner limiting membrane to the retinal pigment epithelium at the fovea) and ganglion cell-inner plexiform layer (GCIPL) thickness were measured using a macular cube 512 × 128 scan. Peripapillary retinal nerve fiber layer (RNFL) thickness was measured using an optic disk cube 200 × 200 scan. All scans were performed by the same experienced examiner (MZ). All of the scan images were reviewed by an experienced ophthalmologist (CW) for further quality control with the following exclusion criteria: (1) poor scan quality (less than 7/10 signal strength index); (2) motion artifacts; (3) inaccurate segmentation; (4) focal signal loss; and (5) blurred images.

Images were analyzed automatically using the AngioPlex OCTA software (version 10.0.0.14618, Carl Zeiss Meditec). Moreover, the FAZ boundaries were carefully reviewed and manually corrected if an obvious error of automated segmentation is observed.

### Data Analysis

One eye with a higher-quality image on the 3 × 3 mm OCTA scan from each subject was selected for the analyses. All data were analyzed using the SPSS 22.0 statistical software package (SPSS, Armonk, NY, United States).

Quantitative variables were described as mean (SD, standard deviation) or median (IOR, interquartile range), while categorical variables were described using frequencies and percentages. The *t*-test was used to evaluate normally distributed data. For non-normally distributed data, we used the Mann–Whitney *U*-test. Correlations between OCTA parameters and other clinical features were evaluated using Spearman correlation analysis. *P* < 0.05 was accepted as statistically different. The area under the receiver operating characteristic curve (AUROC) was calculated to determine the diagnostic accuracy of the analyzed parameters discriminating between PD patients and healthy controls.

## Results

### Clinical Characteristics of Enrolled Patients and Healthy Controls

**FIGURE 1:**
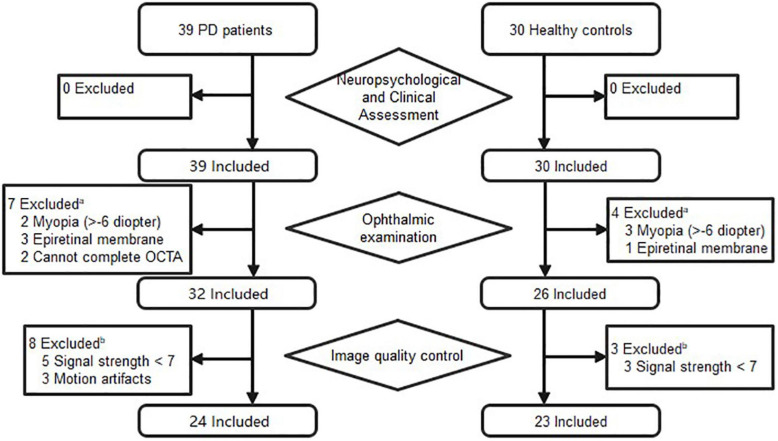
Flow diagram of excluded subjects prior to analysis. ^a^Patients were excluded if one eye had concomitant ocular diseases. ^b^Patients were included if one eye’s images met the inclusion criteria.

A total of 39 patients with a definite diagnosis of idiopathic PD and 30 healthy controls were recruited from the Neurology Department of the First Affiliated Hospital of Sun Yat-sen University between October 2019 and November 2020. After the ophthalmic assessment, seven PD patients and four controls expired, due to concomitant ocular diseases or non-cooperation. Eight patients and three controls were excluded because of insufficient image quality. Figure 1 details the reasons for exclusion from statistical analyses. Ultimately, 24 patients (24 eyes) and 23 controls (23 eyes) were included in the analyses. The PD patients had a mean age of 65.88 years, and 75.0% were male. The healthy controls had a mean age of 63.43 years, and 47.8% were male. There was no significant difference between PD patients and controls with regard to age, and sex. The mean disease duration of PD patients was 5.3 years, and the mean score of UPDRS III was 26.5. The demographics of PD patients and controls are summarized in Table 1.

**TABLE 1:** Demographic characteristics of all enrolled participants.

Variable	Mean (SD)		
	PD	HC	*P*
	*N* = 24	*N* = 23	
Age (year)	65.88 (6.50)	63.43 (7.11)	0.225
Male sex, No. (%)	18/24 (75.0)	11/23 (47.8)	0.055
PD history (year)	5.3 (4.2)	/	/
UPDRS III rating	26.5 (12.3)	/	/
H&Y rating	2.0 (0.3)	/	/
MMSE	28.5 (1.6)	/	/

*PD, Parkinson’s disease; HC, healthy controls; UPDRS III, Unified Parkinson’s Disease Rating Scale III; H&Y, Hoehn and Yahr; MMSE, Mini-Mental State Examination.*

### Visual Function of Enrolled Patients and Healthy Controls

**FIGURE 2:**
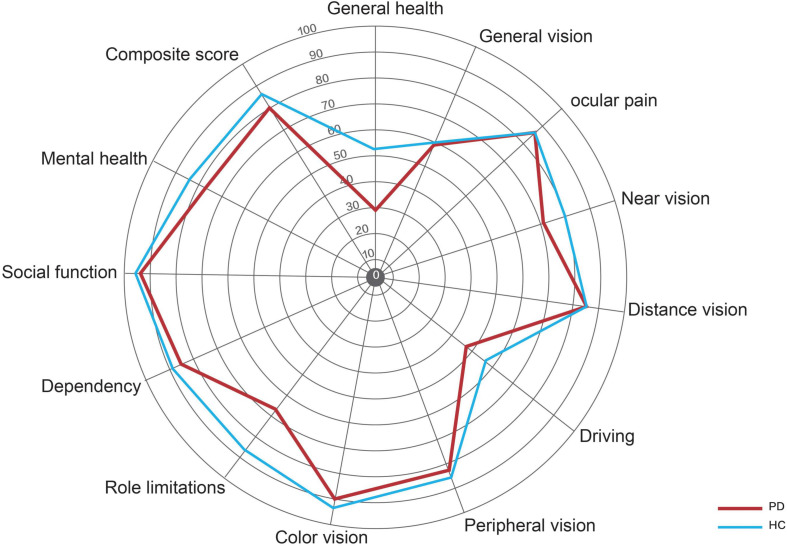
Radar graph representing the mean scores for the various scales in the NEI VFQ-25. The red line represents the patients with PD, and the blue line represents the healthy controls.

There were no significant differences in BCVA and IOP between PD patients and healthy controls. Compared with healthy controls, P100 latency was significantly longer in patients with PD (113.3 ± 14.7 ms vs. 107.6 ± 12.6 ms, *P* = 0.041), whereas P100 amplitude was not significantly different between the two groups (Table 2). The NEI VFQ-25 scores were significantly worse in PD patients for the composite score (80 ± 10 vs. 84 ± 13, *P* = 0.031) and 3 of 12 subscales, including general health (29 ± 16 vs. 52 ± 20, *P* < 0.001), near vision (71 ± 17 vs. 80 ± 22, *P* = 0.037), and role limitations (68 ± 27 vs. 87 ± 19, *P* = 0.008) (Figure 2). The correlations between the general health subscale and other subscales of NEI VFQ-25 were undertaken in order to analyze whether the general health status of PD had an impact on the vision-related quality-of-life assessments. No correlation was found between the general health subscale and other subscales in NEI VFQ-25.

**TABLE 2:** Ophthalmologic and VEP information of all enrolled participants.

Variable	PD (*N* = 24)		HC (*N* = 23)		*P*
	Mean (SD)	Median (range)	Mean (SD)	Median (range)	
BCVA (LogMAR)	0.088 (0.122)	0.000 (0.000–0.191)	0.097 (0.117)	0.097 (0.000–0.097)	0.591
IOP (mmHg)	14.7 (2.4)	14 (13–16)	15.6 (2.8)	16 (13–17)	0.287
P100 latency (ms)	113.3 (14.7)	113.0 (106.0–116.5)	107.6 (12.6)	105.0 (101.5–110.5)	0.041^a^
P100 amplitude (μV)	6.6 (5.1)	5.5 (3.4–8.6)	5.6 (3.9)	4.7 (3.1–7.7)	0.597
NEI VFQ-25 composite score	80 (10)	79 (76–88)	84 (13)	89 (81–93)	0.031^a^

*VEP, visual evoked potential; PD, Parkinson’s disease; HC, healthy controls; BCVA, best-corrected visual acuity; LogMAR, logarithm of the minimum angle of resolution; IOP, intraocular pressure; NEI VFQ-25, 25-item National Eye Institute Visual Function Questionnaire.*

*^a^*P* < 0.05.*

### Macular and Peripapillary Microvascular Density Parameters

**FIGURE 3:**
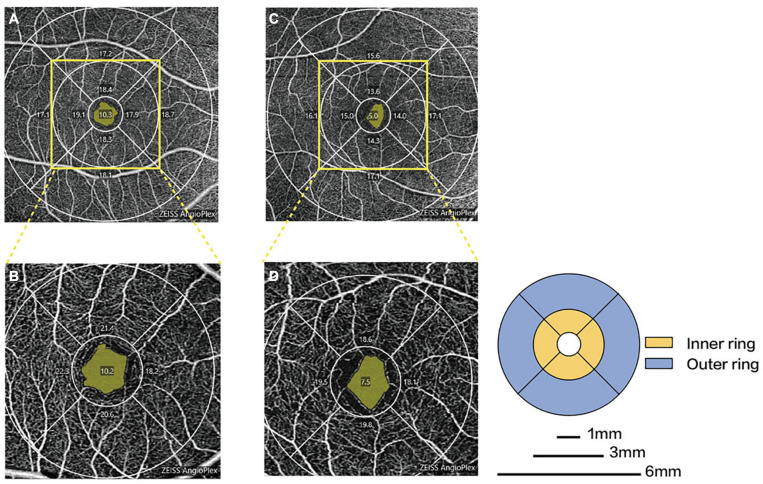
Representative OCTA images in patients with PD and healthy controls. Macular microvascular density of healthy controls **(A)** and PD patients **(C)** in the 6-mm-diameter region. Macular microvascular density of healthy controls **(B)** and PD patients **(D)** in the 3-mm-diameter region.

Parkinson’s disease patients had significantly lower macular VD than healthy controls in the inner superior sector (13.6 ± 4.1 mm vs. 15.6 ± 3.9 mm^–1^, *P* = 0.030) of the 6-mm-diameter region (Figures 3A,C and Supplementary Table 1). Health controls had higher signal strength than PD patients in the 6 × 6 mm scan centered on the macular (8.7 ± 1.0 vs. 7.7 ± 1.1, *P* = 0.008). In the 6-mm-diameter region centered on the ONH, the peripapillary VD in the outer superior sector (16.9 ± 2.2 mm^–1^ vs. 18.6 ± 1.4 mm^–1^, *P* = 0.003) of PD patients was significantly lower than those of controls, while the differences in other regions were not statistically significant (Supplementary Table 2). In the 3-mm-diameter region, macular VD was significantly decreased in all sectors in PD patients compared with healthy controls (full area: 16.0 ± 3.0 mm^–1^ vs. 18.0 ± 2.0 mm^–1^, *P* = 0.010) (Figures 3B,D and Table 3). The signal strength between the two groups was not significantly different in the 3 × 3 mm scan centered on the macular or the 6 × 6 mm scan centered on the ONH (all *P* > 0.05). No significant difference was seen in the FAZ area between the two groups (0.31 ± 0.10 mm^2^ vs. 0.28 ± 0.10 mm^2^, *P* = 0.464).

**TABLE 3:** Comparison of macular microvascular density and FAZ area between the PD patients and healthy controls in the 3-mm-diameter region.

Variable	PD (*N* = 24)		HC (*N* = 23)		*P*
	Mean (SD)	Median (range)	Mean (SD)	Median (range)	
Nasal (mm^–1^)	17.4 (3.5)	17.8 (15.0–19.3)	19.4 (2.6)	19.5 (17.6–21.3)	0.029^a^
Temporal (mm^–1^)	17.2 (3.6)	17.8 (16.0–20.0)	19.7 (1.9)	19.5 (18.6–21.5)	0.009^a^
Superior (mm^–1^)	17.1 (4.1)	18.1 (14.5–20.3)	19.4 (2.6)	19.8 (17.6–21.8)	0.049^a^
Inferior (mm^–1^)	17.1 (3.1)	17.9 (14.9–19.4)	18.8 (2.5)	19.1 (17.1–20.9)	0.049^a^
Central (mm^–1^)	6.6 (2.6)	6.7 (4.9–8.6)	8.2 (2.3)	7.9 (6.5–9.9)	0.032^a^
Inner ring (mm^–1^)	17.2 (3.2)	17.3 (15.9–19.4)	19.3 (2.1)	19.4 (17.9–21.1)	0.011^a^
Full area (mm^–1^)	16.0 (3.0)	16.1 (14.5–18.2)	18.0 (2.0)	18.4 (16.5–19.7)	0.010^a^
FAZ area (mm^2^)	0.31 (0.10)	0.31 (0.22–0.37)	0.28 (0.10)	0.28 (0.23–0.37)	0.464

*FAZ, foveal avascular zone; PD, Parkinson’s disease; HC, healthy controls.*

*^a^*P* < 0.05.*

### CST, RNFL, and GCIPL Thicknesses

The differences between the RNFL thickness (average: 93.4 ± 10.7 μm vs. 98.0 ± 9.4 μm, *P* = 0.128) and CST (average: 272.8 ± 10.3 μm vs. 274.7 ± 11.0 μm, *P* = 0.534) of the two groups were not statistically significant (Supplementary Tables 3, 4). No significant thinning was shown in the GCIPL thickness of PD patients when compared with the healthy controls (average: 80.5 ± 5.6 μm vs. 82.3 ± 6.2 μm, *P* = 0.303) (Supplementary Table 5).

### Correlations Among Visual Function and Disease Duration, Severity, and Cognition in PD Patients

A significant negative correlation was shown between disease severity using the UPDRS III and composite scores of NEI VFQ-25 (*r* = −0.587, *P* = 0.005). The BCVA, VEP P100 latency, and amplitude were not statistically associated with disease duration, severity, and MMSE scores. Spearman correlation coefficients and their corresponding *P*-values are listed in Table 4.

**TABLE 4:** Correlations between visual function and clinical data in PD patients.

	BCVA (LogMAR)	P100 latency	P100 amplitude	Composite score
PD history (year)	−0.188 (0.391)	0.243 (0.288)	0.418 (0.060)	0.051 (0.816)
UPDRS III rating	−0.085 (0.715)	−0.253 (0.282)	−0.267 (0.255)	−0.587 (0.005^a^)
MMSE	−0.048 (0.828)	−0.098 (0.673)	−0.089 (0.701)	−0.023 (0.919)

*PD, Parkinson’s disease; UPDRS III, Unified Parkinson’s Disease Rating Scale III; MMSE, Mini-Mental State Examination; BCVA, best-corrected visual acuity; LogMAR, logarithm of the minimum angle of resolution.*

*^a^*P* < 0.05.*

### Correlations Between Microvascular Density and Clinical Data in PD Patients

The nasal sector and inner ring of macular VD were negatively correlated with disease duration (*r* = −0.442, *P* = 0.035; *r* = −0.431, and *P* = 0.040; respectively), while no significant correlation between disease severity and cognitive function and macular VD of 3-mm-diameter region in PD group (Supplementary Table 6). P100 latency was negatively correlated with the nasal and superior sectors of macular VD in 3-mm-diameter region (*r* = −0.328, *P* = 0.030; *r* = −0.302, and *P* = 0.047, respectively). There was no significant correlation between visual function parameters and other regions of macular VD in the 3-mm-diameter region (Table 5).

**TABLE 5:** Correlations between visual function and macular microvascular density in the 3-mm-diameter region.

	Nasal	Temporal	Superior	Inferior	Central	Inner ring	Full area
BCVA (LogMAR)	0.004 (0.979)	−0.109 (0.466)	−0.060 (0.689)	−0.009 (0.952)	0.066 (0.658)	−0.074 (0.620)	−0.046 (0.761)
P100 latency (ms)	−0.328 (0.030^a^)	−0.113 (0.465)	−0.302 (0.047^a^)	−0.154 (0.318)	−0.176 (0.253)	−0.257 (0.092)	−0.288 (0.058)
P100 amplitude (μV)	0.022 (0.888)	0.243 (0.112)	0.168 (0.275)	0.271 (0.075)	0.169 (0.273)	0.154 (0.319)	0.193 (0.209)
Composite score	0.256 (0.082)	0.175 (0.240)	0.247 (0.094)	0.024 (0.872)	−0.028 (0.849)	0.226 (0.127)	0.194 (0.191)

*BCVA, best-corrected visual acuity; LogMAR, logarithm of the minimum angle of resolution.*

*^a^*P* < 0.05.*

### Diagnostic Abilities of Macular Microvascular Density Indices

**FIGURE 4:**
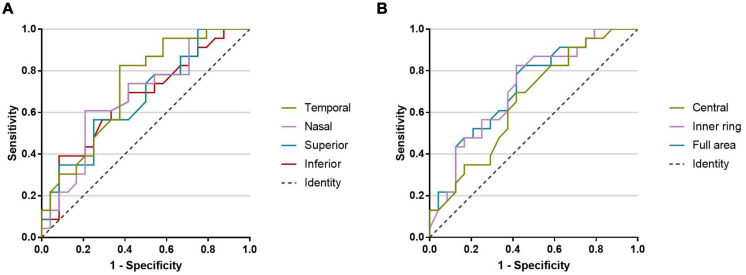
The AUROCs for discriminating PD patients from healthy controls. **(A)** Macular microvascular density of the 3-mm-diameter region: temporal sector (0.723), nasal sector (0.678), superior sector (0.668), and inferior sector (0.668). **(B)** Macular microvascular density of the 3-mm-diameter region: central sector (0.655), inner ring (0.716), and full area (0.720).

The AUROCs of macular VD in the 3-mm-diameter region for discriminating PD patients from healthy controls were highest for the temporal sector (0.723), followed by the full area (0.720), inner ring (0.716), nasal sector (0.678), superior sector (0.668), inferior sector (0.668), and central sector (0.655) (Figure 4).

## Discussion

In this study, we observed that PD patients had no significant visual decline compared with healthy controls but experienced a worse vision-related quality of life, which implied that visual impairment existed in PD patients. Compared with those in healthy controls, prolonged P100 latency and decreased VD in both the macula and ONH were observed in PD patients, but there were no differences in retinal structure between the two groups. In addition, there was a significant correlation between visual impairment and retinal VD. Therefore, our results indicated that the macular microvascular alterations may occur earlier than the changes in retinal structure and may associate with visual impairment.

Symptoms of visual dysfunction have been widely reported and may contribute to the overall disabilities in PD patients (Uc et al., 2005). NEI VFQ-25 is a questionnaire developed by the National Eye Institute used for measuring the self-reported, vision-targeted health status of people with chronic eye diseases (Mangione et al., 2001). Although there was no significant difference in BCVA between the two groups, PD patients had significantly lower near vision, role limitations, and overall composite scores of NEI VFQ-25 compared with healthy controls. Furthermore, the composite score was strongly inversely related to disease severity. These outcomes suggested that PD patients had substantial problems and restrictions in everyday activities due to vision impairment, and worsen along disease progression. It is worth mentioning that the MMSE scores of the PD group were all within the normal range, and the general health score did not correlate with any other subscales. Therefore, it is reasonable to assume that NEI VFQ-25 scores were reliable and not affected by cognitive function, and health status of PD patients. Almer et al. (2012) found that most subscales in NEI VFQ-25 were worse in PD patients than in controls and that near activities seemed to be more greatly affected by disease severity. They suggested that the decline in vision-targeted life quality of PD patients was probably ascribed to the ocular motor function because acuity, color vision, and contrast sensitivity did not vary significantly from those of controls. Compared with those in our study, PD patients in their group had a worse vision-related quality of life. This discrepancy might be ascribed to the difference in the disease course in which the subjects of Almer et al. (2012) had longer disease duration (10.9 ± 6.8 years). A better understanding of the vision-related life quality is important for the optimal care of PD patients, and we strongly encourage PD patients to undergo the assessment of NEI VFQ-25, which is simple, fast, and convenient.

Abnormal VEP in our study also reflected functional impairment in the visual pathway of PD patients. VEP was used as a non-invasive technique to evaluate bioelectrical function of the entire visual pathway from the retina to higher cortical visual pathways. VEP latency was considered to be a more sensitive measure of foveal electrical activity than amplitude and less likely to be affected by dopaminergic drugs. Due to the relatively small individual difference, the P100 latency was commonly used to confirm the abnormalities of the visual pathway. In our study, the P100 latency of VEP was prolonged in patients with PD than in controls, and whereas the difference in P100 amplitude was not significant. This result was consistent with a meta−analysis that contained 20 case–control studies (He et al., 2018). The possible mechanism of VEP abnormalities is that dopaminergic neuron degeneration and decrease in dopamine production and secretion in PD patients may affect the function of the inner plexiform cells and horizontal cells in the retina, disrupting the transmission of visual signals (Büttner et al., 1996). The thinning of intraretinal layers in PD patients as shown by OCT also supports this conclusion to some extent (Garcia-Martin et al., 2014). However, we did not observe such a decrease in the retinal structure, and no significant thinning was shown in RNFL, CST, and GCIPL thicknesses between PD patients and controls in our study. Instead, we found altered retinal microvasculature in PD patients, and microvascular density was lower in PD patients than in controls, particularly in the macular area. As retinal vasculature has embryologic origins similar to cerebral vasculature, it might be postulated that these two shared a common vascular pathophysiology (Gariano and Gardner, 2005; Nadal et al., 2020). Cerebral small-vessel disease was proven to be associated with incident parkinsonism and may be an underlying etiology (Bohnen and Muller, 2016). A recent study has demonstrated that α-synuclein was deposited in the retinal ganglion cell layer as well as the vessel wall of retina arteries in transgenic animal models of PD (Price et al., 2016). Few studies have investigated the relationship between visual function and OCTA in PD. Intriguingly, we found a negative correlation between P100 latency and nasal, and superior sectors of macular VD in the 3-mm-diameter region. It can be speculated that the deposition of α-synuclein in intraretinal layers and retinal vessels, leading to abnormal VEP and microvasculature alterations, and ultimately results in visual dysfunction in PD patients. Likely, the nasal and superior sectors of retinal vessels might be the most affected site, leading to a potential change in the visual pathway at an early stage in PD patients. However, this conjecture must be corroborated by further research.

We also found that PD patients had decreased macular VD in all sectors of the 3-mm-diameter region compared with healthy subjects through the 3 × 3 mm scan without significant difference in signal strength. However, within the same matching area in the 6 × 6 mm scan, macular VD decreased only in the superior sector. We suspect that the discrepancy might be derived from the different scan protocols. In the 6 × 6 mm scan pattern, a total number of 350 B-scans were sampled and repeated twice in the vertical dimension, with each B-scan having 350 A-scans in the horizontal dimension. As for the 3 × 3 mm scan, there were 245 A-scans in each B-scan along the horizontal dimension, and 245 B-scans were repeated four times at each location. Therefore, the 3 × 3 mm scan pattern provides a denser sampling spacing (12.2 μm) than the 6 × 6 mm scanning pattern (17.1 μm), as well as better lateral resolution (Rosenfeld et al., 2016). This enables the 3 × 3 mm scan pattern to acquire more detailed information of the microvasculature and has a greater ability to discriminate the capillaries. Furthermore, a previous study has proved that a direct comparison between the two is not possible, given the different resolutions (Xiao et al., 2020). Considering that capillary deficits could be the earliest change of retinal vessels in PD patients, the 3 × 3 mm scan may more accurately reflect the foveal microvasculature in PD patients. Several studies have adopted OCTA as the main evaluation method to quantify the retinal microvasculature in PD patients. Decreased macular VD and perfusion density, as well as choroidal structural changes, were found in patients with PD in a recent study with a large sample size of 137 healthy controls, and 69 patients. Likewise, no significant difference was found in the retinal structure (Robbins et al., 2020). Furthermore, decreased retinal microvascular density was found only in the SCP but not in the deep capillary plexus in a study by Kwapong et al. (2018). However, in contrast to our results, they detected retinal thinning in PD patients, which was correlated with SCP, and suggesting that retinal microvascular abnormalities may contribute to the neurodegeneration in PD patients (Kwapong et al., 2018). Shi et al. (2020) used three different algorithms to quantify the retinal capillaries based on the OCTA images. They found that the retinal capillary skeleton and perfusion densities and capillary complexity of SCP were significantly lower in PD patients than in healthy controls (Shi et al., 2020). Unlike our results, in the study by Rascunà et al. (2020) no significant difference in microvascular density was observed between PD patients and healthy controls. These results were variable and inconsistent, possibly due to the study design, and patient selection. Furthermore, a prior study showed that different OCTA devices had different effects on VD measurements in subjects with media opacity, possibly due to different OCTA flow algorithms (Zhang et al., 2020). In addition, VD measurements and the OCTA repeatability were significantly affected by the signal strength (Lee et al., 2019; Yu et al., 2019). Therefore, the type of OCTA devices and the signal strength of OCTA images need to be taken into account when interpreting the results. Although results from these studies differed, most of them supported the notion that macular VD was decreased in PD patients. The details of different studies and their findings are elucidated in Supplementary Table 7. Our results suggested that retinal microvascular changes might precede vision decline and be detected earlier than retinal structure changes in PD patients. Furthermore, macular microvascular density showed diagnostic capacities to distinguish PD patients from healthy controls (AUROCs, ranged from 0.655 to 0.723). Within our study, PD patients were at a mean H&Y stage of 2, which indicated that our patients were in a relatively early stage of the disease. Thus, retinal microvasculature changes show promise as biomarkers for the diagnosis of PD in the early stage.

The present study had several notable advantages. First, we assessed the PD patients’ UPDRS III and H&Y stage in the “off” state, which could more accurately reflect the severity of the disease without the impact of medications. Second, NEI VFQ-25 was used to evaluate vision-related quality of life, which could more closely mirror the actual practice of visual function. Third, different from prior studies, we analyzed the correlation between VEP parameters and retinal microvascular density. Of note is that both of these are objective measurements without subjective bias. However, there are some limitations of this study that merit considerations. The current sample size was small, so the results must be carefully interpreted because some of the detected correlations could be statistical anomalies. Although we assessed the BCVA, one of the primary indices of visual impairment, we did not evaluate specific visual symptoms, such as ocular motor function, contrast sensitivity, color perception, and illusions, which are reported commonly in PD and may also contribute to the lower life quality. Even we excluded the poor quality of images, the healthy group had higher-quality OCTA images than the PD group in the 6 × 6 mm scan centered on the macular, which may cause bias when interpreting these results. Another noteworthy point is that PD patients in the severe stage have difficulty cooperating with the OCTA examination, so we could not acquire the data from this group. However, on the basis of our results, we remain convinced that retinal microvascular density might be valuable in the diagnosing and monitoring for early PD. Future studies are needed to further investigate clinical implications of our findings.

## Conclusion

Our study showed that retinal microvascular density decreased in PD patients and correlated with visual impairment. Retinal microvasculature was altered early even when the visual decline and retinal structure changes are not detectable, and may be a promising diagnostic marker for PD patients.

## Data Availability Statement

The raw data supporting the conclusions of this article will be made available by the authors, without undue reservation.

## Ethics Statement

The studies involving human participants were reviewed and approved by Ethics Committee of the First Affiliated Hospital of Sun Yat-sen University. The patients/participants provided their written informed consent to participate in this study.

## Author Contributions

MZ and LW: study and original manuscript writing. PW, MZ, and LW: conception and design. CW, JY, and TC: data collection. MZ and QH: analysis and interpretation. CW and PW: manuscript revision. PW: funding. All authors contributed to the article and approved the submitted version.

## Conflict of Interest

The authors declare that the research was conducted in the absence of any commercial or financial relationships that could be construed as a potential conflict of interest. The reviewer CL declared a shared affiliation, with no collaboration, with the authors to the handling editor at the time of the review.

## Publisher’s Note

All claims expressed in this article are solely those of the authors and do not necessarily represent those of their affiliated organizations, or those of the publisher, the editors and the reviewers. Any product that may be evaluated in this article, or claim that may be made by its manufacturer, is not guaranteed or endorsed by the publisher.

## Abbreviations

PD: Parkinson’s disease
CNS: central nervous system
OCTA: optical coherence tomography angiography
OCT: optical coherence tomography
VEP: visual evoked potential
MMSE: Mini-Mental State Examination
UPDRS III: Unified Parkinson’s Disease Rating Scale III
H&Y: Hoehn and Yahr
BCVA: best-corrected visual acuity
IOP: intraocular pressure
NEI VFQ-25: 25-item National Eye Institute Visual Function Questionnaire
ONH: optic nerve head
VD: vessel density
SCP: superficial capillary plexus
FAZ: foveal avascular zone
CST: central subfield thickness
GCIPL: ganglion cell-inner plexiform layer
RNFL: retinal nerve fiber layer
SD: standard deviation
IQR: interquartile range
AUROC: the area under receiver operating characteristic curve
LogMAR: logarithm of the minimum angle of resolution.
