# Supplementary material for: Visual Impairments Are Associated With Retinal Microvascular Density in Patients With Parkinson’s Disease
Source: Front Neurosci. 2021 Aug 12;15:718820. doi: 10.3389/fnins.2021.718820 (PMC8406760; doi:10.3389/fnins.2021.718820)
Supplement: Supplementary file 1 [file Data_Sheet_1.docx]

**Supplementary Online Content**

Supplementary Table 1. Comparison of Macular Microvascular Density Between the PD Patients and Healthy Controls in the 6mm Diameter Region

Supplementary Table 2. Comparison of Peripapillary Microvascular Density Between the PD Patients and Healthy Controls in the 6mm Diameter Region

Supplementary Table 3. Comparison of RNFL Thickness Between the PD Patients and Healthy Controls

Supplementary Table 4. Comparison of CST Between the PD Patients and Healthy Controls

Supplementary Table 5. Comparison of the GCIPL Thickness Between Patients with PD and Healthy Controls

Supplementary Table 6. Correlations Between Clinical Data and Macular Microvascular Density of 3mm Diameter Region in PD patients

Supplementary Table 7. Studies using optical coherence tomography angiography in Parkinson’s disease

| Supplementary Table 1. Comparison of Macular Microvascular Density Between the PD Patients and Healthy Controls in the 6mm Diameter Region | | | | | |
| --- | --- | --- | --- | --- | --- |
|  | PD (N=24) | | HC (N=23) | | *P* |
| Variable (mm^-1^) | Mean (SD) | Median (range) | Mean (SD) | Median (range) |  |
| Inner nasal | 13.7 (4.3) | 14.0 (11.5 to 17.0) | 15.1 (4.0) | 16.5 (13.7 to 18.0) | 0.195 |
| Inner temporal | 13.5 (5.0) | 15.5 (8.4 to 16.7) | 15.1 (4.9) | 16.9 (14.4 to 18.3) | 0.091 |
| Inner superior | 13.6 (4.1) | 15.7 (11.7 to 16.7) | 15.6 (3.9) | 16.5 (14.9 to 18.2) | 0.030^a^ |
| Inner inferior | 14.2 (3.6) | 15.7 (11.5 to 16.9) | 15.4 (4.1) | 17.2 (14.2 to 18.1) | 0.063 |
| Outer nasal | 17.7 (2.3) | 18.2 (17.0 to 19.3) | 17.7 (3.6) | 18.8 (17.0 to 20.0) | 0.373 |
| Outer temporal | 13.4 (4.5) | 15.2 (10.1 to 16.8) | 14.3 (4.0) | 14.9 (13.1 to 17.1) | 0.684 |
| Outer superior | 14.8 (3.3) | 15.9 (11.7 to 17.2) | 15.7 (3.9) | 16.9 (14.8 to 18.0) | 0.160 |
| Outer inferior | 14.9 (3.0) | 16.0 (11.7 to 17.2) | 15.8 (3.6) | 16.7 (15.3 to 18.1) | 0.218 |
| Central | 4.9 (2.7) | 4.6 (2.9 to 6.6) | 6.3 (2.9) | 7.1 (3.4 to 8.3) | 0.089 |
| Inner ring | 13.8 (4.0) | 15.0 (10.6 to 16.4) | 15.3 (4.1) | 17.0 (14.4 to 18.5) | 0.052 |
| Outer ring | 15.2 (3.0) | 16.4 (13.8 to 17.6) | 15.9 (3.6) | 16.6 (14.9 to 18.2) | 0.184 |
| Full area | 14.6 (3.2) | 15.6 (12.8 to 17.1) | 15.5 (3.7) | 16.5 (14.9 to 17.8) | 0.106 |
| Abbreviations: PD, Parkinson’s disease; HC, healthy controls. | | | | | |

^a^: *P*＜0.05.

| Supplementary Table 2. Comparison of Peripapillary Microvascular Density Between the PD Patients and Healthy Controls in the 6mm Diameter Region | | | |
| --- | --- | --- | --- |
|  | Mean (SD) | | *P* |
| Variable (mm^-1^) | PD (N=24) | HC (N=23） |  |
| Inner nasal | 16.1 (3.6) | 17.0 (2.3) | 0.375 |
| Inner temporal | 15.2 (4.3) | 16.2 (3.7) | 0.444 |
| Inner superior | 17.1 (2.7) | 17.4 (2.6) | 0.846 |
| Inner inferior | 17.2 (2.6) | 17.6 (2.0) | 0.846 |
| Outer nasal | 15.0 (3.9) | 16.5 (2.4) | 0.178 |
| Outer temporal | 17.5 (3.2) | 17.6 (2.6) | 0.923 |
| Outer superior | 16.9 (2.2) | 18.6 (1.4) | 0.003^a^ |
| Outer inferior | 17.0 (3.8) | 18.4 (1.8) | 0.190 |
| Central | 3.2 (3.6) | 2.2 (2.1) | 0.706 |
| Inner ring | 16.4 (2.8) | 17.1 (2.3) | 0.437 |
| Outer ring | 16.7 (2.9) | 17.8 (1.7) | 0.215 |
| Full area | 16.2 (2.6) | 17.2 (1.7) | 0.290 |
| Abbreviations: PD, Parkinson’s disease; HC, healthy controls. | | | |
| ^a^: *P* <0.05. |  |  |  |

| Supplementary Table 3. Comparison of RNFL Thickness Between the PD Patients and Healthy Controls | | | |
| --- | --- | --- | --- |
|  | Mean (SD) | | *P* |
| Variable (μm) | PD (N=24) | HC (N=23) |  |
| Average | 93.4 (10.7) | 98.0 (9.4) | 0.128 |
| Nasal | 67.0 (10.2) | 70.0 (8.0) | 0.290 |
| Temporal | 70.4 (17.1) | 71.2 (10.2) | 0.958 |
| Superior | 114.7 (15.8) | 120.0 (19.0) | 0.300 |
| Inferior | 120.9 (20.1) | 130.8 (18.2) | 0.084 |

Abbreviations: RNFL, retinal nerve fiber layer; PD, Parkinson’s disease; HC, healthy controls.

| Supplementary Table 4. Comparison of CST Between the PD Patients and Healthy Controls | | | |
| --- | --- | --- | --- |
|  | Mean (SD) | | *P* |
| Variable (μm) | PD (N=24) | HC (N=23) |  |
| Central | 246.0 (18.3) | 247.7 (13.1) | 0.710 |
| Average | 272.8 (10.3) | 274.7 (11.0) | 0.534 |
| Inner superior | 314.9 (16.8) | 316.5 (11.5) | 0.698 |
| Inner nasal | 318.8 (16.8) | 320.3 (13.2) | 0.734 |
| Inner inferior | 313.7 (16.2) | 314.8 (13.8) | 0.808 |
| Inner temporal | 305.6 (16.7) | 305.0 (11.5) | 0.882 |
| Outer superior | 271.9 (12.0) | 274.7 (12.5) | 0.433 |
| Outer nasal | 290.8 (15.1) | 294.0 (12.9) | 0.432 |
| Outer inferior | 263.2 (14.4) | 262.9 (11.0) | 0.926 |
| Outer temporal | 257.5 (13.7) | 258.0 (10.3) | 0.904 |
| Abbreviations: CFT, central subfield thickness; PD, Parkinson’s disease; HC, healthy controls. | | | |

| Supplementary Table 5. Comparison of the GCIPL Thickness Between Patients with PD and Healthy Controls | | | |
| --- | --- | --- | --- |
|  | Mean (SD) | | *P* |
| Variable (μm) | PD (N=24) | HC (N=23) |  |
| Average | 80.5 (5.6) | 82.3 (6.2) | 0.303 |
| Superior | 80.3 (6.2) | 82.3 (8.0) | 0.331 |
| Inferior | 78.4 (6.3) | 81.6 (6.3) | 0.100 |
| Superonasal | 82.8 (8.4) | 85.2 (7.2) | 0.200 |
| Inferonasal | 80.5 (7.5) | 82.6 (8.0) | 0.387 |
| Superotemporal | 80.0 (6.4) | 80.1 (6.1) | 0.943 |
| Inferotemporal | 81.1 (4.9) | 82.5 (6.5) | 0.432 |
| Abbreviations: GCIPL, ganglion cell-inner plexiform layer; PD, Parkinson’s disease; HC, healthy controls. | | | |

| Supplementary Table 6. Correlations Between Clinical Data and Macular Microvascular Density of 3mm Diameter Region in PD patients | | | | | | | |
| --- | --- | --- | --- | --- | --- | --- | --- |
|  | Nasal | Temporal | Superior | Inferior | Central | Inner ring | Full area |
| PD History (year) | -0.442(0.035^a^) | -0.314(0.145) | -0.294(0.174) | -0.408(0.054) | -0.279(0.198) | -0.431(0.040^a^) | -0.411(0.051) |
| UPDRS III rating | -0.081(0.726) | -0.160(0.489) | -0.195(0.397) | 0.129(0.577) | 0.047(0.840) | 0.053(0.821) | -0.030(0.896) |
| MMSE | -0.072(0.745) | 0.251(0.247) | 0.106(0.630) | -0.099(0.653) | 0.249(0.253) | 0.049(0.825) | 0.089(0.688) |
| Abbreviations: PD, Parkinson’s disease; UPDRS III, Unified Parkinson Disease Rating Scale III; MMSE, Mini-Mental State Examination. | | | | | | | |
| ^a^: *P*＜0.05. |  |  |  |  |  |  |  |

Supplementary Table 7. Studies using optical coherence tomography angiography in Parkinson’s disease.

| Study | Sample size | OCTA type | Mean age (years) | PD history (years) | UPDRS III | HY rating | Macular thickness | RNFL | GCL/GCIPL | FAZ area | Microvascular density of SCP (macula) | Microvascular density of SCP (ONH) | AUROC |
| --- | --- | --- | --- | --- | --- | --- | --- | --- | --- | --- | --- | --- | --- |
| (Kwapong et al., 2018) | P-38, C-28 | SD-OCTA, Optovue | 61.18±5.74 | 3.84±2.80 | NS | NA | Thinner in the superior, nasal, and inferior quadrants | Thinner in the inner ring of inferior quadrant | Thinner in all but the temporal inner ring and the central region | NS | Decreased in all 4 quadrants (3mm) | NS | NS |
| (Zou et al., 2020) | P-35, C-35 | SD-OCTA, Zeiss | 61.86±5.46 | 3.2±2.0 | NS | 2（1-2） | Thinner  (average) | Thinner in temporal quadrant | Thinner  (average) | No diff | Decreased in central, inner ring and full area (6mm) | NS | 0.618-0.756 |
| (Robbins et al., 2020) | P-69, C-137 | SD-OCTA, Zeiss | 71.1±7.0 | NS | NS | NS | No diff | No diff | No diff | No diff | Decreased in inner ring and full area (6mm) | NS | 0.50-0.63 |
| (Rascunà et al., 2020) | P-21, C-17 | SD-OCTA, Optovue | 61.5±6.5 | 2.3±1.2 | 25.0±6.9 | 1.9±0.4 | NA | Thinner  (average) | Thinner  (average) | NA | No diff (3mm) | NA | NS |
| (Shi et al., 2020) | P-25, C-25 | SD-OCTA, Optovue | 61.9±7.6 | 3.7±2.4 | NS | 2.2±1.0 | Thinner  (average) | No diff | Thinner  (average) | NA | Decreased in all 4 quadrants (3mm)^a^ | NS | NS |
| Our study | P-24, C-23 | SD-OCTA, Zeiss | 65.88±6.50 | 5.3±4.2 | 26.5±12.3 | 2.0±0.3 | No diff | No diff | No diff | No diff | Decreased in inner superior sector(6mm); Decreased in all 4 quadrants (3mm) | Decreased in outer superior (6mm) | 0.655-0.723 |

Abbreviations: P, Patients; C, Controls; SD-OCTA, spectral domain-optical coherence tomography angiography; UPDRS III, Unified Parkinson Disease Rating Scale III; H&Y, Hoehn and Yahr; RNFL, retinal nerve fiber layer; GCL, ganglion cell layer; GCIPL, ganglion cell-inner plexiform layer; FAZ, foveal avascular zone; SCP, superficial capillary plexus; ONH, optic nerve head; AUROC, the area under receiver operating characteristic curve; diff, difference; NA, not available; NS, not studied.

^a^ retinal capillary skeleton density.
